# Supplementary material for: Etiological and epidemiological features of acute respiratory infections in China
Source: Nat Commun. 2021 Aug 18;12:5026. doi: 10.1038/s41467-021-25120-6 (PMC8373954; doi:10.1038/s41467-021-25120-6)
Supplement: Supplementary file 1 — Supplementary Information [file 41467_2021_25120_MOESM1_ESM.pdf]

Supplementary Information

Supplement to: Etiological and epidemiological features of acute respiratory infections in China

Supplementary methods

Data Collection and Statistical Analysis

Detailed demographic and clinical data on cases and laboratory results of specimens were collected by staff of sentinel hospitals and laboratories through a standardized case reporting form, and entered on a weekly basis into an online data management system established by the China CDC.

Descriptive statistics included frequency analysis for categorical variables, medians and interquartile ranges for continuous variables. Pearson's Chi square test or Fisher's exact test were performed to compare categorical variables between groups. Cochran-Armitage trend test was used to analysis age-specific trends in detection rates.

The Join-Point regression (JPR) model was used to describe trends that was not constant over ages. It allows for evaluation of statistically significant changes (join points) in trends. If the age(s) when changes in the trend occur (join points) were found to be statistically significant, then linear regression techniques could be used to estimate the regression parameters. Linear trends were estimated by using annual percentage change (APC) by using the natural log-linear model (ln(y) = xb). A positive value of APC indicated an increasing trend, while negative ones referred to a decreased trend.

The Join-Point regression model for the observations, (x_1, y_1), ..., (x_n, y_n), where $x_1 \leqslant ... \leqslant x_n$ without loss of generality, may be written as

$$E[y|x] = \beta_0+\beta_1 x+\delta_1(x-\tau_1)^{+}+\ldots+\delta_k(x-\tau_k)^{+}$$

where the τ_k 's are the unknown join-points and $\alpha^{+}=\alpha$ for $\alpha>0$ and 0 otherwise.[1]

Linear trends were estimated by using annual percentage change (APC) which is calculated as $APC_i=[Exp(b_i)-1)]\times100$,[2] where b_i represents the slope of the period segment. Average annual percent change (AAPC) is a summary measure of the trend over a prespecified fixed interval. It allows us to use a single number to describe the average APCs over a period of multiple years.

To infer virus-virus, bacteria-bacteria and virus-bacteria interactions at the level of individual hosts, we applied multi-stage logistic regressions to avoid selection bias and obtain robust conclusions to the greatest extent. For the first step, we conducted logistic regressions only with one pathogen as explanatory variable. For each study pathogen (Y), another 'non-Y' pathogens was used as explanatory variable 'X' in turn. The age, sex, illness severity (with or without pneumonia, duration from symptom onset to clinic access) and disease seasonality (month of the year, categorical variable) of the patient were used as covariates for adjustment. By this way, all the 91 pairwise interactions of 13 pathogens (8 viruses and 5 bacteria, we excluded *L. pneumophila, C. pneumoniae* and *GAS* from analysis) between each other were revealed as 'interactions without adjusting for multi-pathogens' (positive interactions with significance were highlighted in red and negative interactions with significance were highlighted in blue in T Supplementary Table 3). For the second stage, results from only13524 cases with all 17 pathogens tested were used for logistic regressions. For each study pathogen (Y), all the remaining 'non-Y' pathogens was used as explanatory variable 'X', and the regressions were performed with every pathogen regarded as independent variables in turn, as these data do not allow for inferences of the directionality of interactions. The age, sex, illness severity and disease seasonality of the patients were similarly adjusted. In our results, adjustment for multiple comparisons were not performed.[3] [4] The interactions of 'X' and 'Y' were considered as significant when significant interaction with

each other was achieved in bidirectional manner. The results disclosed 30 pairs of statistically significant interactions among multi-pathogens (highlighted with green color in the Supplementary Table 4). For the third stage, the significant results from two step logistic regressions were aligned and those consistent were considered as the final robust results.

Supplementary Table 1. Demographic and epidemiological characteristics of patients with acute respiratory infection (ARI) in China mainland, 2009–18.

	All cases (N=231107)	Any virus tested (n=183011)	Any bacteria tested (n=84214)	All virus tested (n=110058)	All bacteria tested (n=26757)	All pathogens tested (n=13524)
**Sex**						
Male	122409 (52.97)	96212 (52.57)	44772 (53.16)	53068 (48.22)	17095 (63.89)	8536 (63.12)
Female	108698 (47.03)	86799 (47.43)	39442 (46.84)	56990 (51.78)	9662 (36.11)	4988 (36.88)
**Age group**						
Children (<5 years)	83701 (36.22)	69213 (37.82)	29611 (35.16)	41921 (38.09)	10517 (39.31)	6207 (45.90)
School-age children (5–17 years)	36815 (15.93)	30810 (16.84)	9143 (10.86)	17663 (16.05)	2121 (7.93)	1155 (8.54)
Adult (18–59 years)	74379 (32.18)	59352 (32.43)	25324 (30.07)	33288 (30.25)	6498 (24.29)	2923 (21.61)
Older people (≥60 years)	36212 (15.67)	23636 (12.92)	20136 (23.91)	17186 (15.62)	7621 (28.48)	3239 (23.95)
**Case type**						
Inpatients	124108 (53.70)	94260 (51.51)	60238 (71.53)	68577 (62.31)	23001 (85.96)	11556 (85.45)
Outpatients	106999 (46.30)	88751 (48.49)	23976 (28.47)	41481 (37.69)	3756 (14.04)	1968 (14.55)
**Clinical type**						
Pneumonia cases	46631 (20.18)	37020 (20.23)	23169 (27.51)	25014 (22.73)	9395 (35.11)	5900 (43.63)
Fatal cases	581 (0.43)	323 (0.18)	171 (0.2)	279 (0.25)	126 (0.47)	98 (0.72)

Data are n (%) unless otherwise indicated. Percentages may not total 100 because of rounding.

Supplementary Fig. 1. Annual number of provinces, cities, sentinel hospitals/ reference laboratories and acute respiratory infection (ARI) patients in the active surveillance study in China, 2009–19.

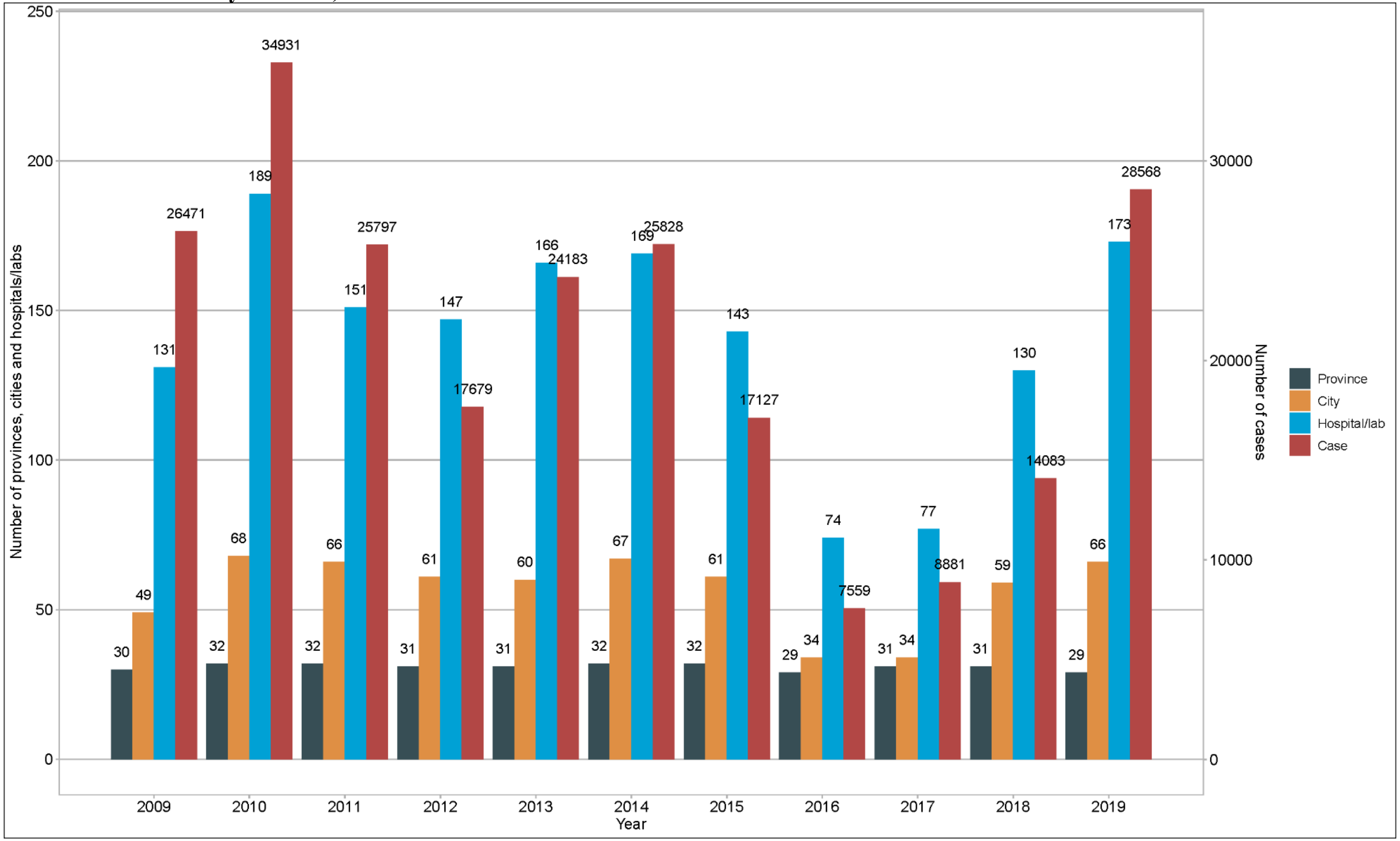

Supplementary Table 2. Pathogen spectrum of patients with ARIs and pneumonia in mainland China, 2009–2019.

	Pneumonia								Non-pneumonia							
		Children		School-age children		Adults		Older people		Children		School-age children		Adults		Older people
**Virus** #																
**IFV***	5	823 (9.77)	2	214 (18.58)	1	435 (39.08)	1	531 (40.69)	3	2648 (16.5)	1	1884 (38.95)	1	4866 (55.76)	1	1470 (42.07)
IFV-A	-	495 (60.15)	-	99 (46.26)	-	275 (63.22)		352 (66.29)		1516 (57.25)		939 (49.84)		2863 (75.18)		960 (65.31)
IFV-B	-	217 (26.37)	-	92 (42.99)	-	101 (23.22)		122 (22.98)		820 (30.97)		745 (39.54)		1328 (19.56)		315 (21.43)
IFV-C	-	6 (0.73)	-	1 (0.47)	-	11 (2.53)		6 (1.13)		28 (1.06)		3 (0.16)		33 (0.08)		32 (2.18)
IFV-untyped	-	105 (12.76)	-	22 (10.28)	-	48 (11.03)		51 (9.60)		284 (10.73)		197 (10.46)		642 (5.17)		163 (11.09)
**RSV***	1	2363 (28.05)	5	125 (10.85)	6	55 (4.94)	5	102 (7.82)	1	3926 (24.46)	5	360 (7.44)	6	393 (4.5)	5	255 (7.3)
RSV-A	-	1137 (48.1)	-	72 (57.6)	-	25 (45.5)		28 (27.4)		1791 (45.6)		133 (36.9)		182 (46.3)		81 (31.8)
RSV-B	-	916 (38.8)	-	36 (28.8)	-	15 (27.3)		22 (21.6)		1155 (29.4)		103 (28.6)		139 (35.4)		108 (42.4)
RSV-untyped	-	310 (13.1)	-	17 (13.6)	-	15 (27.3)		52 (51.0)		980 (25.0)		124 (34.4)		72 (18.3)		66 (25.9)
**HRV**	2	1540 (18.28)	1	267 (23.18)	2	201 (18.06)	2	225 (17.24)	2	2710 (16.88)	3	767 (15.86)	2	1188 (13.61)	2	622 (17.8)
**HPIV***	3	1433 (17.01)	4	166 (14.41)	3	130 (11.68)	3	157 (12.03)	4	2438 (15.19)	4	460 (9.51)	4	706 (8.09)	3	432 (12.36)
HPIV-1	-	184 (12.84)	-	36 (21.69)	-	17 (13.08)		23 (14.65)		409 (16.78)		116 (25.22)		108 (15.3)		61 (14.1)
HPIV-2	-	113 (7.89)	-	31 (18.67)	-	35 (26.92)		35 (22.29)		356 (14.60)		109 (23.70)		196 (27.8)		125 (28.9)
HPIV-3	-	977 (68.18)	-	84 (50.60)	-	54 (41.54)		81 (51.59)		1453 (59.60)		178 (38.70)		299 (42.4)		183 (42.4)
HPIV-4	-	156 (10.89)	-	15 (9.04)	-	19 (14.62)		16 (10.19)		200 (8.20)		50 (10.87)		103 (14.6)		63 (14.6)
HPIV-untyped	-	3 (0.21)	-	0 (0)	-	5 (3.85)		2 (1.27)		20 (0.82)		7 (1.52)		0 (0)		0 (0)
**HAdV**	4	834 (9.9)	3	179 (15.54)	4	120 (10.78)	7	48 (3.68)	5	1780 (11.09)	2	776 (16.04)	3	726 (8.32)	6	180 (5.15)
**HCoV**	8	304 (3.61)	7	69 (5.99)	5	100 (8.98)	4	157 (12.03)	6	902 (5.62)	6	302 (6.24)	5	472 (5.41)	4	313 (8.96)
**HBoV**	6	714 (8.47)	6	79 (6.86)	8	15 (1.35)	8	17 (1.3)	7	899 (5.6)	8	139 (2.87)	8	155 (1.78)	8	64 (1.83)
**HMPV**	7	414 (4.91)	8	53 (4.6)	7	57 (5.12)	6	68 (5.21)	8	748 (4.66)	7	149 (3.08)	7	221 (2.53)	7	158 (4.52)
**Bacterium** #																
S. pneumoniae	1	470 (37.15)	2	51 (11.78)	2	147 (22.79)	1	205 (25.37)	1	639 (39.64)	2	98 (29.34)	1	217 (24.41)	1	273 (26.38)
M. pneumoniae	3	168 (13.28)	1	315 (72.75)	1	178 (27.6)	6	30 (3.71)	2	372 (23.08)	1	120 (35.93)	6	17 (1.64)	6	17 (1.64)
H. influenzae	2	302 (23.87)	3	21 (4.85)	5	77 (11.94)	4	111 (13.74)	3	283 (17.56)	3	49 (14.67)	4	141 (13.62)	4	141 (13.62)
K. pneumoniae	5	116 (9.17)	5	11 (2.54)	4	80 (12.4)	3	165 (20.42)	5	98 (6.08)	4	20 (5.99)	2	165 (18.56)	3	221 (21.35)
P. aeruginosa	6	48 (3.79)	4	13 (3)	3	95 (14.73)	2	204 (25.25)	7	28 (1.74)	8	7 (2.1)	3	158 (17.77)	2	250 (24.15)
S. aureus	4	142 (11.23)	6	10 (2.31)	6	44 (6.82)	5	79 (9.78)	4	137 (8.5)	5	17 (5.09)	5	85 (9.56)	5	108 (10.43)
C. pneumoniae	7	17 (1.34)	7	8 (1.85)	7	11 (1.71)	8	5 (0.62)	6	31 (1.92)	6	12 (3.59)	7	15 (1.45)	7	15 (1.45)
L. pneumophila	8	1 (0.08)	8	2 (0.46)	9	6 (0.93)	7	6 (0.74)	8	22 (1.36)	7	9 (2.69)	9	4 (0.39)	9	4 (0.39)
GAS	9	1 (0.08)	9	2 (0.46)	8	7 (1.09)	9	3 (0.37)	9	2 (0.12)	9	2 (0.6)	8	6 (0.58)	8	6 (0.58)

Data are n (%) unless otherwise indicated. Proportions may not total 100 because of rounding. # The pneumonia or non-pneumonia cases with all 8 viruses tested were used to determine

the spectrum of viruses and the pneumonia or non-pneumonia cases with all 9 bacteria tested were used to determine the spectrum of bacteria. The rank and proportion of positive cases (proportion) was given in the table. For each pathogen, the numerator was its number of positive cases and denominator was the total number of positive cases for all pathogens. * The proportion of subtypes for IFV, RSV and HPIV indicate the proportion within each pathogen.

Supplementary Fig. 2 Codetection pattern in ARI patients by age groups in China, 2009–18. Panel A: children's pattern. Panel B: school-age children' pattern. Panel C: adults' pattern. Panel D: older people's pattern. Codetection rates were calculated pairwise. For pathogen, 'X' and 'Y', numerator was total number of patients coinfected both 'X' and 'Y' and the denominator were total number of patients who were both tested 'X' and 'Y'.

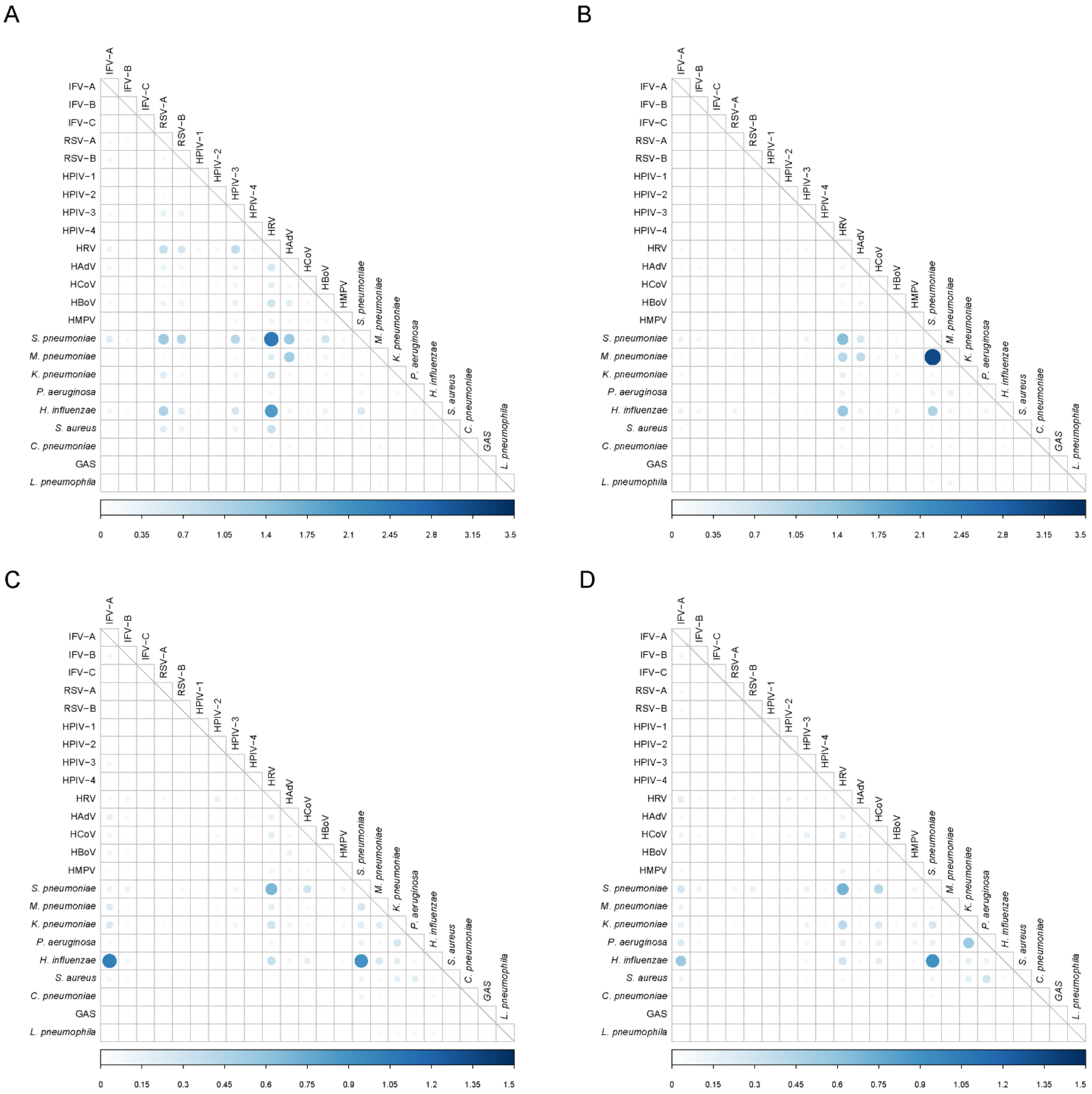

Supplementary Table 3. Interaction of all the detected pathogens in pairwise by using logistic regression.

	IFV-A	IFV-B	IFV-C	RSV-A	RSV-B	HPIV-1	HPIV-2	HPIV-3	HPIV-4	HRV	HAdV	HCoV	HBoV	HMPV	*S. p*	*M. p*	*K. p*	*P. a*	*H. i*
**IFV-A**	-									-									
**IFV-B**	0.19																		
**IFV-C**	0.16	0.51																	
**RSV-A**	0.32	0.65	1.27																
**RSV-B**	0.62	0.61	1.24	0.3															
**HPIV-1**	0.48	0.68	0	1.26	1.41														
**HPIV-2**	0.43	0.43	13.91	2.24	0.75	2.9													
**HPIV-3**	0.57	0.47	2.61	1.34	1.66	0.94	1.17												
**HPIV-4**	0.61	2.31	0	1.58	1.23	1.23	1.67	0.52											
**HRV**	0.47	0.39	1.75	1.25	1.38	1.25	2.91	1.42	1.42										
**HAdV**	0.36	0.47	0.24	0.9	0.63	1.08	1.47	0.93	2.12	1.13									
**HCoV**	0.45	0.60	1.19	1.19	1.22	2.4	8.61	2.06	1.09	1.82	1.47								
**HBoV**	0.91	1.21	0	1.41	1.44	1.4	1.73	1.78	2.18	2.04	3.03	1.53							
**HMPV**	0.38	0.43	1.82	0.62	0.69	1.58	2.53	1.25	1.24	1.51	1.24	2.44	2.15						
S. p	0.81	1.32	0.45	1.14	1.15	0.87	0.89	1.12	1.54	1.21	1.68	1.84	1.12	1.17					
M. p	0.27	0.15	0	0.27	0.27	0.86	0.31	0.44	0.44	0.68	2.6	0.83	1.07	0.97	5.78				
K. p	0.80	1.00	0.87	2.06	0.96	0.63	0.89	1.06	1.02	1.31	0.87	1.41	1.19	2.21	0.69	0.62			
P. a	0.82	0.97	0	1.19	0.79	0.68	0.24	0.68	0.24	0.72	1.01	0.52	2.04	0.97	0.37	0.77	1.32		
H. i	0.89	1.33	0.93	1.59	0.9	1.66	1.44	1.07	0.61	1.13	0.52	1.29	0.84	0.58	4.52	0.47	0.67	0.46	
S. a	0.83	1.63	0	1.76	1.22	0.95	0.58	1.27	1.24	0.96	0.23	0.61	0.81	0.61	0.33	0.3	0.87	1.23	0.64

S. p: S. pneumoniae; M. p: M. pneumonia; K. p: K. pneumonia; P. a: P. aeruginosa; H. i: H. influenzae; S. a: S. aureus.

Table only shows the odd ratio of pathogens. Red indicates positive interactions with two-sided $p<0.05$ and blue indicates negative ones with two-sided $p<0.05$. Column names indicates response variables and row names indicates explanatory variables. The interactions with statistical significance were so called "interactions without adjusting for multi-pathogens".

Supplementary Table 4. Interaction of all the detected pathogens in pairwise by using multivariate logistic regression.

	IFV-A	IFV-B	IFV-C	RSV-A	RSV-B	HPIV-1	HPIV-2	HPIV-3	HPIV-4	HRV	HAdV	HCoV	HBoV	HMPV	***S. p***	***M. p***	***K. p***	***P. a***	***H. i***	***S. a***
**IFV-A**	-	0.55	2.16	0.48	0.71	0.38	0.83	0.54	0.63	0.6	0.67	0.56	1.48	0.64	1.09	0.07	0.79	0.76	0.78	0.7
**IFV-B**	0.54	-	0	0.87	1.66	2.43	0	0.79	0.76	0.68	0.85	0.78	0.98	0.43	1.16	0.36	1.04	1.31	0.85	1.31
**IFV-C**	2.21	0	-	0.79	0	0	0	4.44	0	1.27	0	0	0	0	2.63	0	4.83	0	0	0
**RSV-A**	0.44	0.73	0.79	-	0.15	0.61	0.42	0.79	1.3	0.89	0.45	0.78	0.85	0.66	1.04	0.3	1.43	0.6	1.34	1.36
**RSV-B**	0.64	1.43	0	0.14	-	0.53	0.77	0.95	0.81	0.84	0.73	0.84	1.14	0.84	1.26	0.25	0.93	0.75	0.74	1.02
**HPIV-1**	0.39	2.35	0	0.58	0.55	-	3.6	0.35	1.25	1.17	0.34	1.77	1.29	0.78	0.87	0.71	0.62	0.98	1.41	0.73
**HPIV-2**	0.88	0	0	0.47	0.86	3.5	-	0.96	1.63	0.93	2.79	2.06	0.86	0.5	1.15	0	0.81	0	1.48	0.81
**HPIV-3**	0.58	0.76	3.97	0.7	0.9	0.35	1.03	-	0.26	1.15	1.15	3.17	1.41	0.88	1.11	0.41	0.94	0.64	0.89	1.02
**HPIV-4**	0.62	0.71	0	1.22	0.85	1.13	1.66	0.24	-	1.07	0.73	1.58	0.32	0.3	1.39	0.15	0.9	0.33	0.37	1.15
**HRV**	0.6	0.67	1.11	0.84	0.79	1.15	0.94	1.16	1.07	-	0.97	1.1	1.39	1.07	1.2	0.56	1.34	0.72	1.06	0.97
**HAdV**	0.66	0.82	0	0.46	0.74	0.34	2.78	1.14	0.7	0.99	-	1.04	1.43	0.98	0.7	1.06	0.31	0.75	0.66	0.28
**HCoV**	0.55	0.78	0	0.85	1.01	1.83	2.13	3.32	1.56	1.16	1.04	-	1.41	1.82	1.86	0.61	1.56	0.39	1.09	0.51
**HBoV**	1.59	1.04	0	0.88	1.22	1.33	0.85	1.47	0.35	1.43	1.43	1.37	-	1.27	0.96	0.65	0.88	1.71	0.63	0.57
**HMPV**	0.65	0.42	0	0.63	0.76	0.77	0.49	0.87	0.29	1.05	0.96	1.78	1.14	-	1.02	0.33	2.19	0.82	0.53	0.55
S. p	1.12	1.17	2.39	1.04	1.26	0.9	1.12	1.05	1.31	1.18	0.68	1.84	0.89	1.02	-	0.48	0.65	0.5	3.61	0.27
M. p	0.07	0.34	0	0.4	0.32	0.79	0	0.48	0.19	0.61	1.09	0.66	0.78	0.36	0.47	-	0.88	1.13	0.62	0.38
K. p	0.78	1.01	4.85	1.32	0.91	0.63	0.79	1.03	0.9	1.38	0.31	1.55	0.89	2.19	0.66	0.88	-	3.75	0.82	1.24
P. a	0.69	1.25	0	0.74	0.84	0.98	0	0.69	0.29	0.71	0.74	0.41	1.69	0.81	0.56	1.14	3.78	-	1.06	3.2
H. i	0.82	0.88	0	1.19	0.7	1.39	1.46	0.91	0.39	1.04	0.63	1	0.66	0.52	3.58	0.63	0.8	0.89	-	0.69
S. a	0.68	1.27	0	1.2	0.97	0.72	0.83	0.99	1.12	0.97	0.27	0.52	0.53	0.54	0.27	0.39	1.26	2.97	0.74	-

S. p: S. pneumoniae; M. p: M. pneumonia; K. p: K. pneumonia; P. a: P. aeruginosa; H. i: H. influenzae; S. a: S. aureus.

Table only shows the odd ratio of pathogens. Red indicates positive interactions with two-sided $p<0.05$ and blue indicates negative ones with $p<0.05$. Column names indicates response variables and row names indicates explanatory variables. Green background indicates the pairwise interaction between 'X' and 'Y' as both response and explanatory variables reached consistent. The interactions with statistical significance were so called "interactions adjusting for multi-pathogens".

Supplementary Table 5. Positive rate of respiratory pathogens by age, gender and case type in patients with acute respiratory infections (ARIs) in China, 2009–19.

	All Cases	Sex		P value	Age Group				P value	Case Type		P value
		Male	Female		Children	School-age children	Adults	Older people		Inpatients	Outpatients	
**IFV**	28463 (15.6)	14306 (14.93)	14157 (16.4)	<0.001	6518 (9.47)	5757 (18.75)	13135 (22.20)	3053 (12.94)	<0.001	10258 (10.94)	18205 (20.55)	<0.001
IFV-A	*18588 (9.64)*	*9648 (10.21)*	*8940 (9.09)*	*<0.001*	*3616 (5.05)*	*3598 (11.28)*	*9215 (14.49)*	*2159 (8.41)*	*<0.001*	*5607 (5.33)*	*12981 (14.83)*	*<0.001*
IFV-B	*5941 (3.08)*	*2926 (3.1)*	*3015 (3.07)*	*0.715*	*1603 (2.24)*	*1445 (4.53)*	*2318 (3.65)*	*575 (2.24)*	*<0.001*	*2199 (2.09)*	*3742 (4.27)*	*<0.001*
IFV-C	*191 (0.1)*	*94 (0.1)*	*97 (0.1)*	*1.000*	*52 (0.07)*	*15 (0.05)*	*78 (0.12)*	*46 (0.18)*	*<0.001*	*129 (0.12)*	*62 (0.07)*	*<0.001*
**HRV**	7614 (6.8)	4167 (7.81)	3447 (6.0)	<0.001	4309 (10.15)	1043 (5.85)	1404 (4.19)	858 (4.94)	<0.001	5675 (8.15)	1939 (4.67)	<0.001
**RSV**	11973 (6.9)	7130 (7.84)	4843 (5.9)	<0.001	9920 (14.59)	842 (2.91)	734 (1.37)	477 (2.09)	<0.001	9486 (10.27)	2487 (3.07)	<0.001
RSV-A	*4961 (2.71)*	*2944 (3.29)*	*2017 (2.15)*	*<0.001*	*4121 (5.91)*	*321 (1.06)*	*347 (0.6)*	*172 (0.69)*	*<0.001*	*4011 (3.91)*	*950 (1.18)*	*<0.001*
RSV-B	*3690 (2.01)*	*2151 (2.41)*	*1539 (1.64)*	*<0.001*	*3062 (4.39)*	*219 (0.72)*	*246 (0.42)*	*163 (0.65)*	*<0.001*	*2925 (2.85)*	*765 (0.95)*	*<0.001*
**HPIV**	8711 (5.0)	4860 (5.34)	3851 (4.7)	<0.001	5814 (8.56)	1036 (3.58)	1171 (2.18)	690 (3.03)	<0.001	6113 (6.63)	2598 (3.20)	<0.001
HPIV-1	*1584 (0.86)*	*827 (0.91)*	*757 (0.8)*	*0.009*	*1019 (1.43)*	*225 (0.74)*	*225 (0.39)*	*115 (0.46)*	*<0.001*	*1014 (0.97)*	*570 (0.7)*	*<0.001*
HPIV-2	*1238 (0.67)*	*631 (0.7)*	*607 (0.64)*	*0.157*	*580 (0.81)*	*188 (0.62)*	*284 (0.49)*	*186 (0.75)*	*<0.001*	*916 (0.88)*	*322 (0.4)*	*<0.001*
HPIV-3	*4751 (2.57)*	*2636 (2.91)*	*2115 (2.24)*	*<0.001*	*3490 (4.9)*	*420 (1.38)*	*518 (0.89)*	*323 (1.29)*	*<0.001*	*3589 (3.45)*	*1162 (1.44)*	*<0.001*
HPIV-4	*782 (0.42)*	*382 (0.42)*	*400 (0.42)*	*0.996*	*457 (0.64)*	*89 (0.29)*	*144 (0.25)*	*92 (0.37)*	*<0.001*	*516 (0.5)*	*266 (0.33)*	*<0.001*
**HAdV**	7411 (4.3)	3909 (4.27)	3502 (4.2)	0.712	4326 (6.36)	1428 (4.92)	1361 (2.51)	296 (1.30)	<0.001	4853 (5.23)	2558 (3.14)	<0.001
**HCoV**	3438 (2.0)	1879 (2.13)	1559 (1.9)	0.006	1665 (2.59)	481 (1.69)	745 (1.39)	547 (2.42)	<0.001	2141 (2.43)	1297 (1.61)	<0.001
**HBoV**	2933 (1.9)	1708 (2.11)	1225 (1.6)	<0.001	2313 (3.77)	273 (1.08)	253 (0.52)	94 (0.43)	<0.001	2312 (2.63)	621 (0.90)	<0.001
**HMPV**	2676 (1.6)	1376 (1.56)	1300 (1.6)	0.342	1688 (2.63)	290 (1.02)	428 (0.80)	270 (1.19)	<0.001	1762 (1.99)	914 (1.14)	<0.001
S. pneumoniae	3755 (7.3)	1750 (7.41)	1750 (7.4)	0.343	1830 (11.14)	279 (7.10)	743 (4.93)	903 (5.62)	<0.001	3348 (7.81)	407 (4.73)	<0.001
M. pneumoniae	4104 (5.8)	2180 (6.23)	2180 (6.2)	<0.001	1931 (7.44)	1188 (14.47)	800 (3.79)	185 (1.16)	<0.001	3545 (6.91)	559 (2.81)	<0.001
K. pneumoniae	2278 (4.6)	900 (3.93)	900 (3.9)	<0.001	513 (3.25)	81 (2.14)	712 (5.00)	972 (6.24)	<0.001	1755 (4.24)	523 (6.50)	<0.001
P. aeruginosa	2081 (4.2)	931 (4.07)	931 (4.1)	0.130	245 (1.56)	84 (2.22)	713 (5.01)	1039 (6.67)	<0.001	1635 (3.96)	446 (5.56)	<0.001
H. influenzae	1968 (4.0)	1007 (4.39)	1007 (4.4)	<0.001	846 (5.39)	142 (3.75)	471 (3.28)	509 (3.23)	<0.001	1678 (4.03)	290 (3.63)	0.099
S. aureus	1620 (3.3)	664 (2.90)	664 (2.9)	<0.001	538 (3.41)	87 (2.30)	467 (3.29)	528 (3.39)	0.005	1214 (2.94)	406 (5.08)	<0.001
C. pneumoniae	352 (0.5)	152 (0.45)	152 (0.4)	0.034	171 (0.67)	57 (0.72)	74 (0.37)	50 (0.32)	<0.001	292 (0.58)	60 (0.31)	<0.001
GAS	136 (0.3)	47 (0.21)	47 (0.2)	0.007	37 (0.24)	12 (0.32)	48 (0.34)	39 (0.25)	0.317	98 (0.24)	38 (0.48)	<0.001
L. pneumophila	89 (0.2)	43 (0.24)	43 (0.2)	1.000	34 (0.26)	16 (0.53)	24 (0.23)	15 (0.14)	0.001	82 (0.25)	7 (0.15)	0.245

Chi square test or Fisher’s exact test were used for comparisons among different groups.
The statistical test used was two-sided.
For each pathogen, the numerator was the number of positive cases and denominator was the number of patients testing for the corresponding pathogen.

Supplementary Fig. 3. The Join-Point regression of detection rates of each virus subtype over patient's age in an acute respiratory infection surveillance study, China, 2009–19. Each panel shows the regression result of each bacterium. For each panel, red point indicates detection rate of cases at each age (year) and the colored segments are the fitted curves. Legends give the Annual Percent Change (APC) value of each fitted curve for each virus. * indicates that the APC is significantly from zero at alpha =0.05 level. IFV-C and HPIV-4 were not conducted Join-Point regression due to low sample size. The grey bars indicate the number of patients tested for each pathogen.

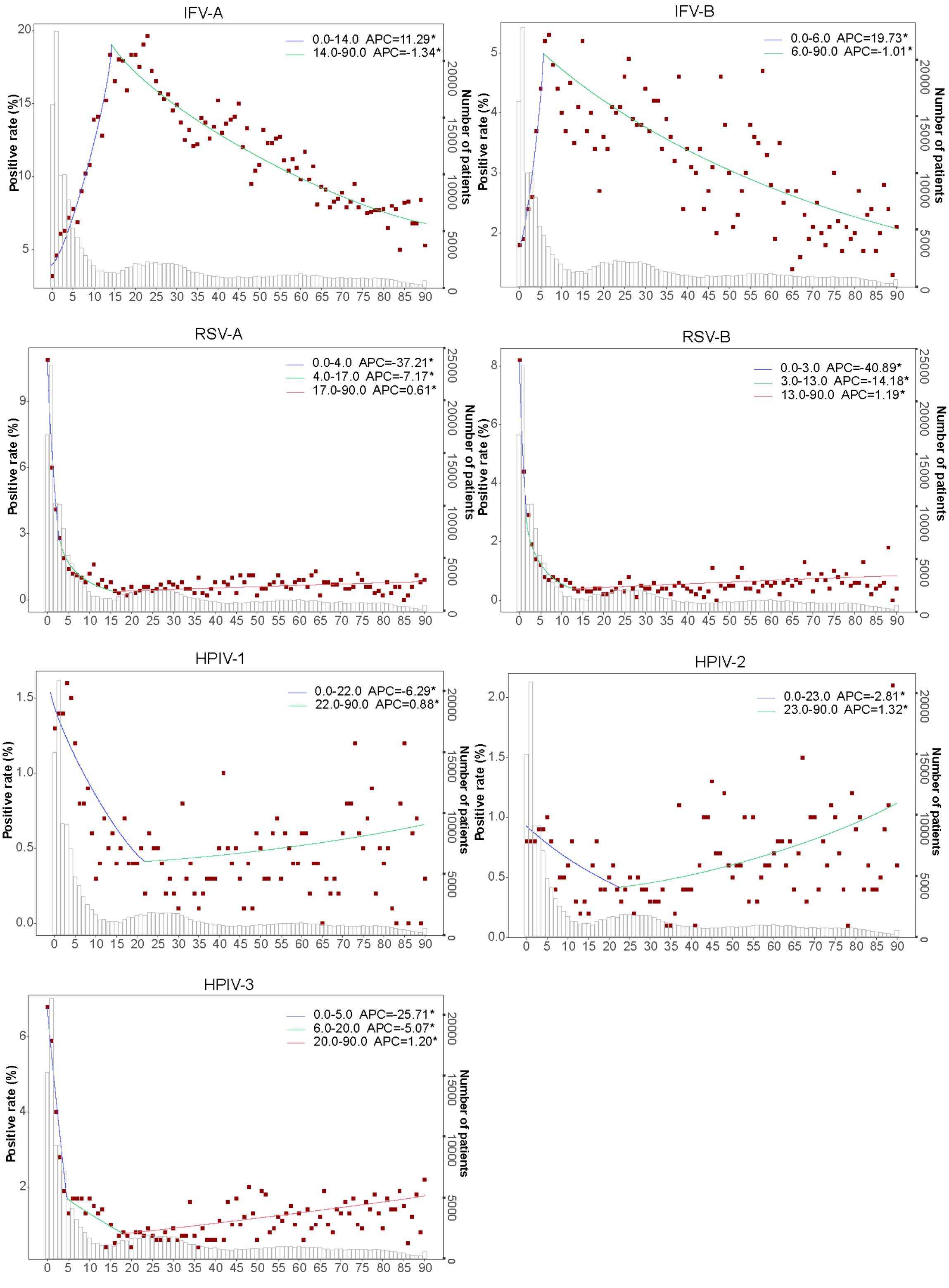

Supplementary Fig. 4. The Join-Point regression of detection rates of each bacterium over patient's age in an acute respiratory infection surveillance study, China, 2009–19. Each panel shows the regression result of each bacterium. For each panel, red point indicates detection rate of cases at each age (year) and the colored segments are the fitted curves. Legends give the Annual Percent Change (APC) value of each fitted curve for each virus. * indicates that the APC is significantly from zero at alpha =0.05 level. The grey bars indicate the number of patients tested for each pathogen.

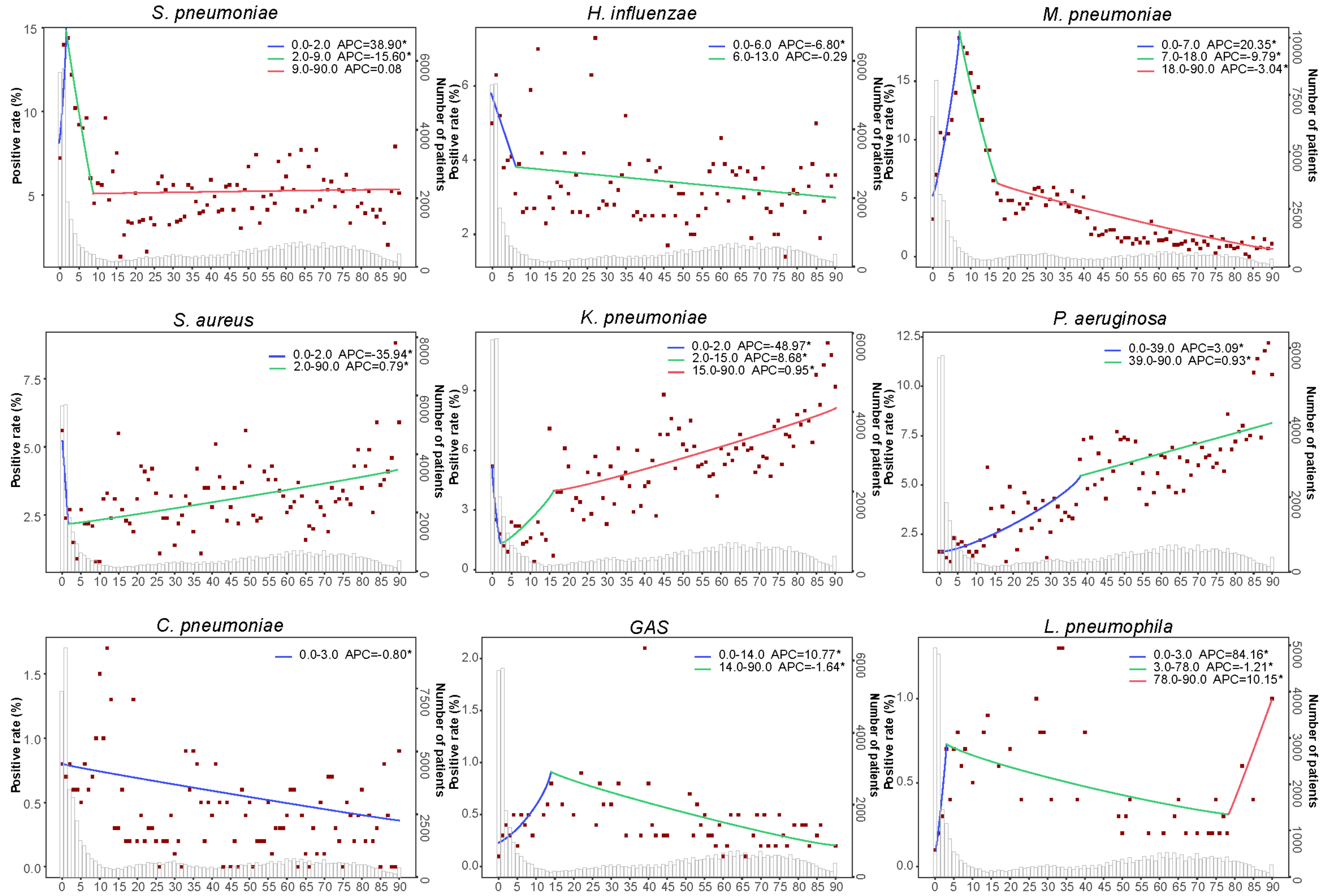

Supplementary Fig. 5. Annual patterns of detected respiratory pathogens. A) Positive rate of viruses. B) Positive rate of bacteria. C) Positive rate of influenza virus (IFV) types. D) Positive rate of respiratory syncytial virus (RSV) types. E) Positive rate of human parainfluenza virus (HPIV) types.

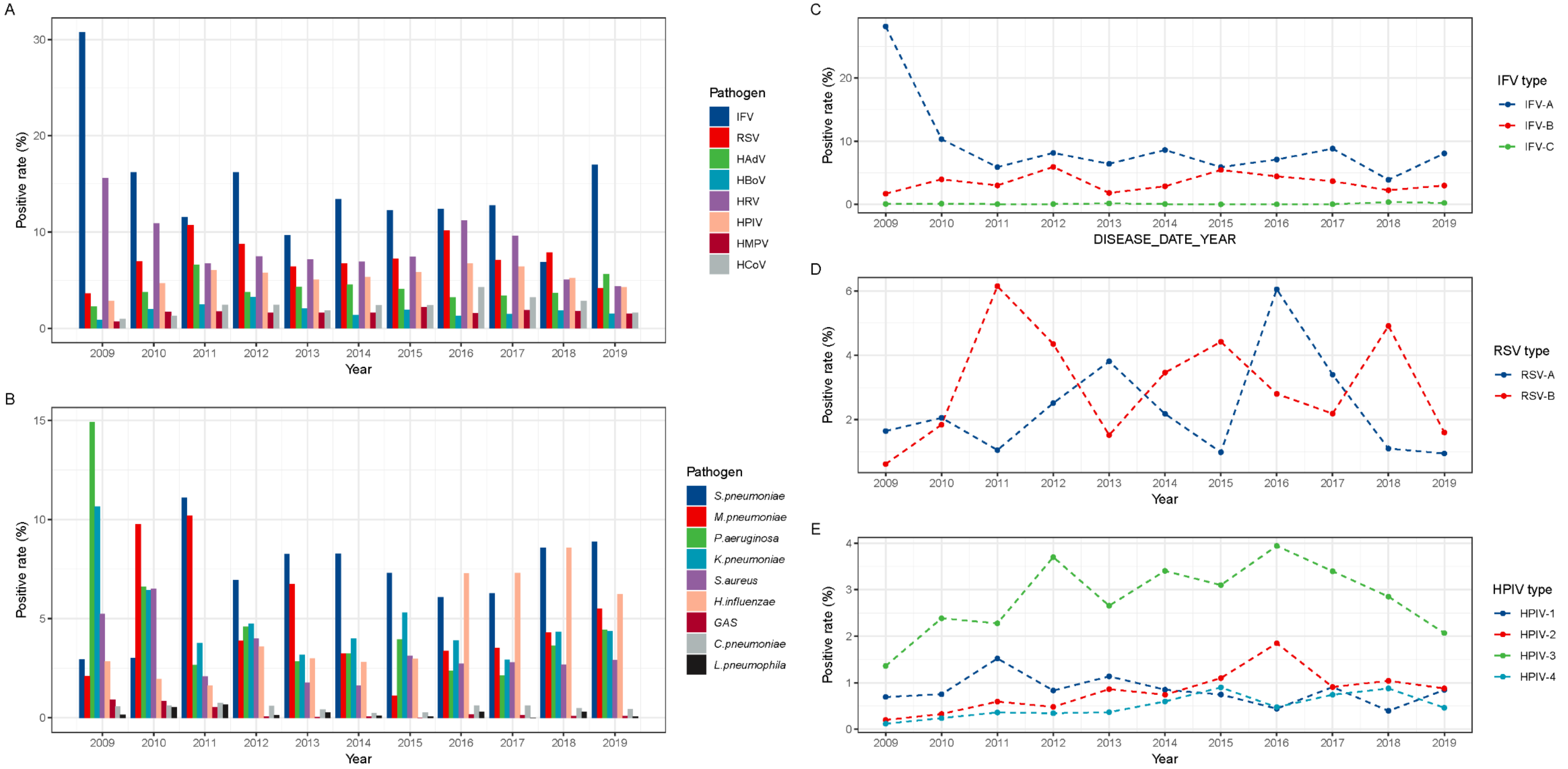

Supplementary Fig. 6. The spatial grouping of provinces into the north of the mainland of China (blue) and south of the mainland of China (red).

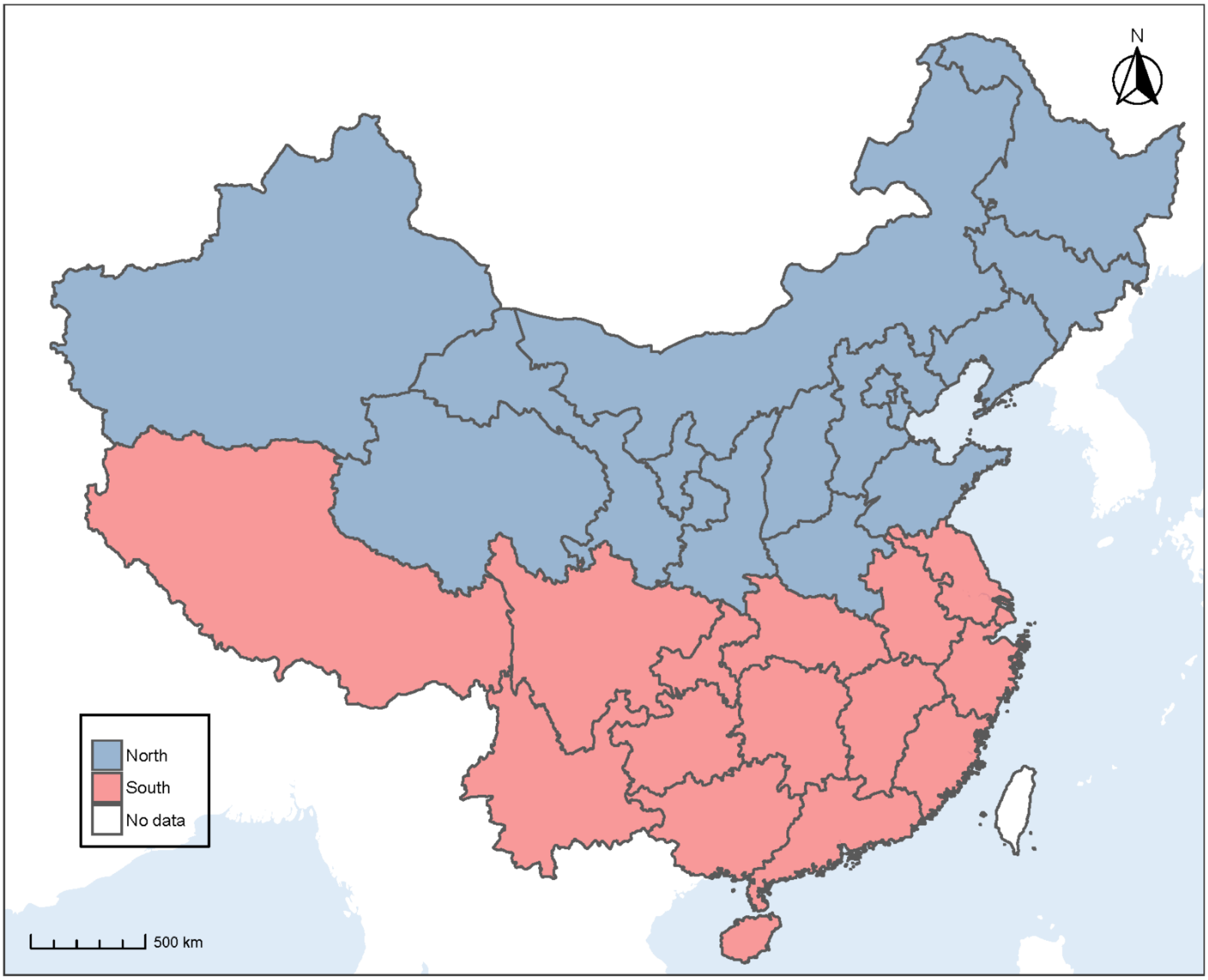

Supplementary Fig. 7. Seasonality pattern of respiratory viruses by southern and northern China. Each bar indicates the monthly average positive rate of corresponding pathogen. The red bars indicate southern China and the blue bars indicate northern China.

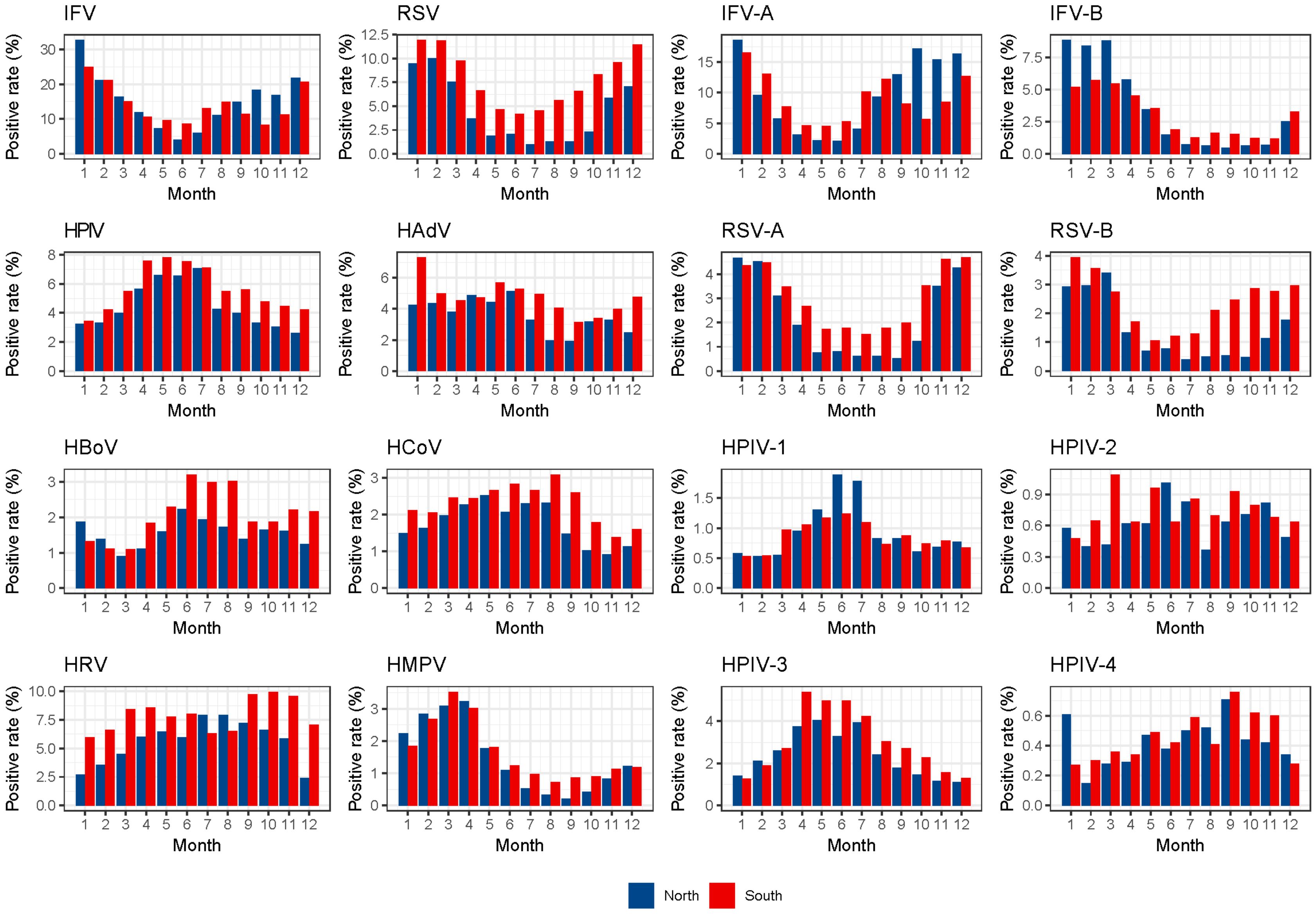

Supplementary Fig. 8. The flowchart of laboratory tests for bacteria.

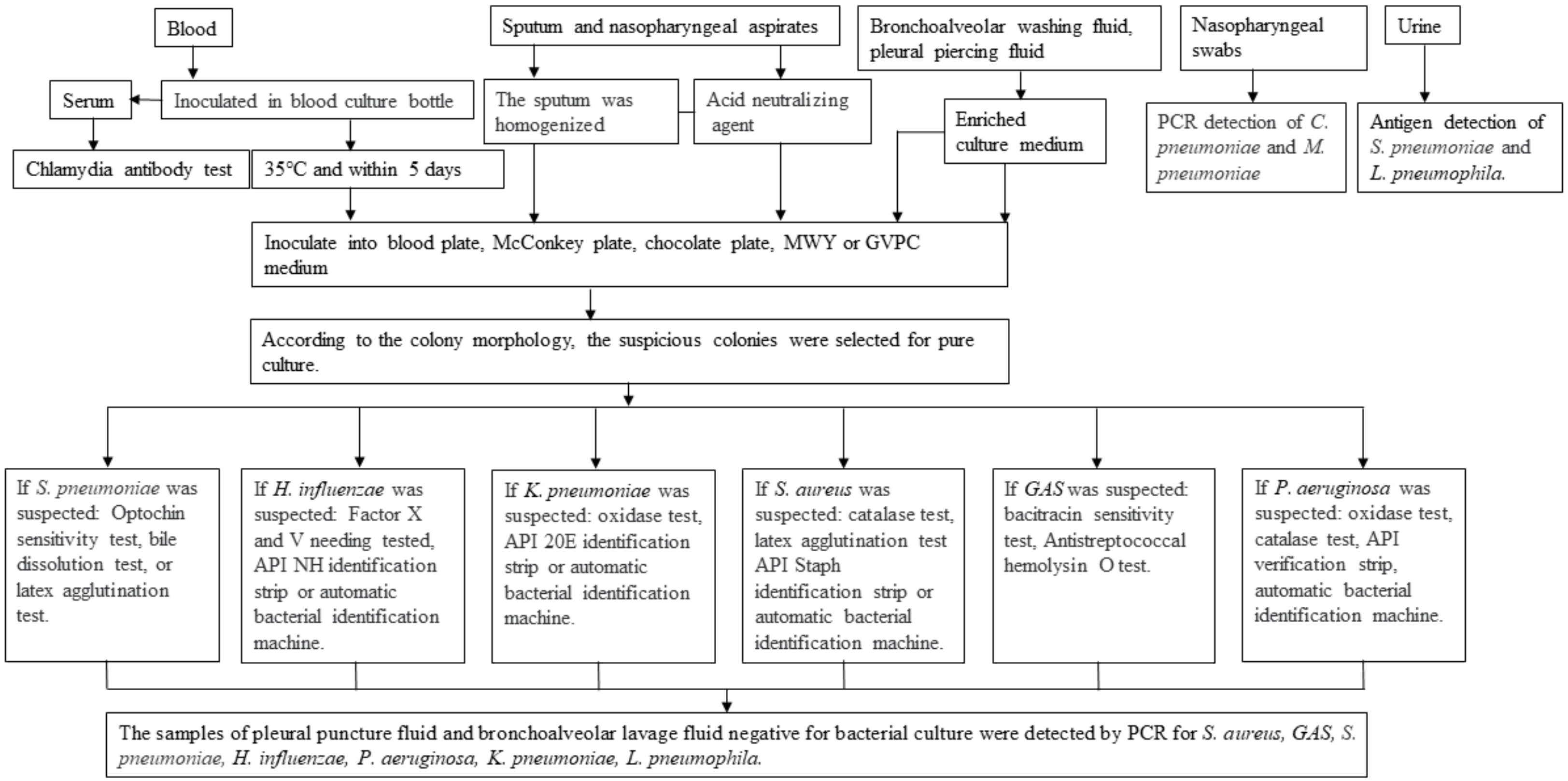

Supplementary Fig. 9. The flowchart of laboratory tests for viruses.

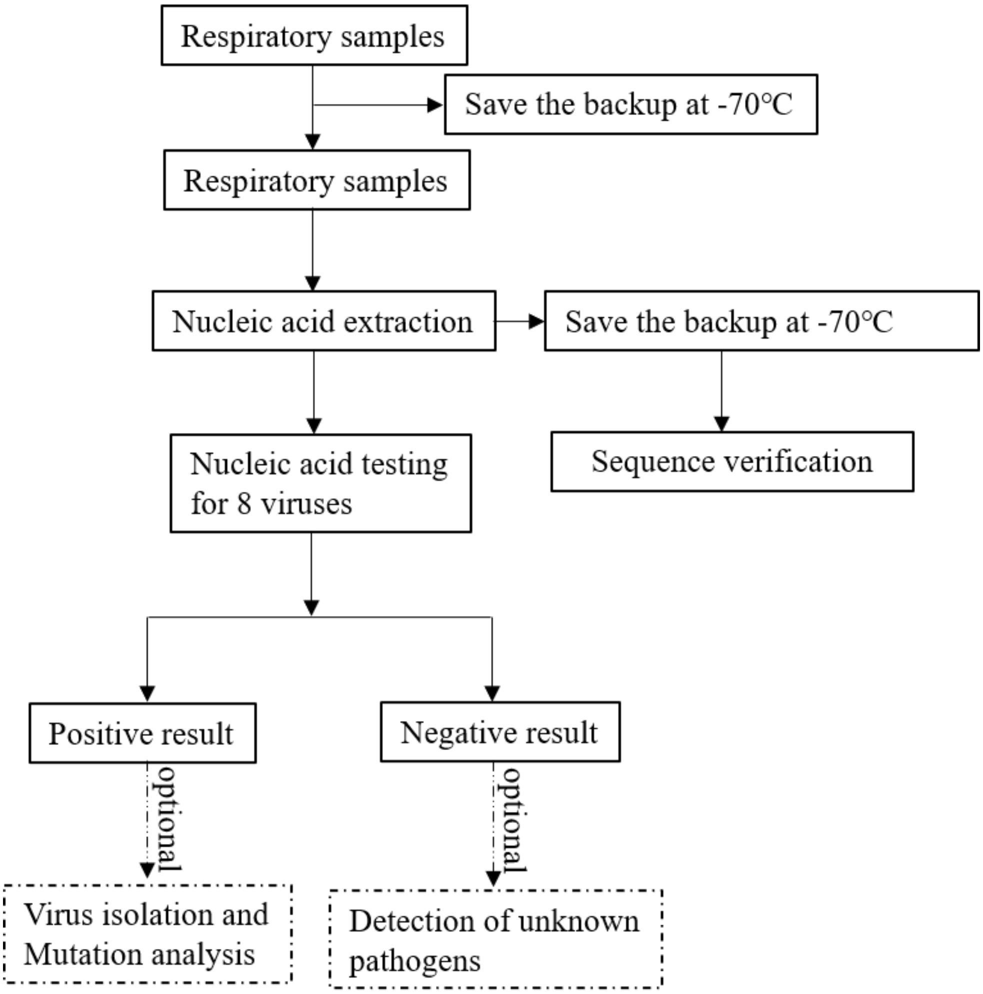

Supplementary Table 6. Primers and sequence information for PCR used in acute respiratory infection surveillance of China, 2009-2019.

Supplementary Table 6-1. Primers and sequence information used for characterizing respiratory viruses.

Pathogen	Method	Primers	Sequence (5'-3')	Gene amplification	Amplicon size	Reference
HBoV	Touchdown PCR	AK-VP-F1	CGCCGTGGCTCCTGCTCT	VP1/VP2	611 bp	5
		AK-VP-R1	TGTTCGCCATCACAAAAGATGTG			
		AK-VP-F2	GGCTCCTGCTCTAGGAAATAAAGAG		576 bp	
		AK-VP-R2	CCTGCTGTTAGGTCGTTGTTGTATGT			
HRV	RT-PCR	RV-F1	CTCCGGCCCCTGAATRYGGCTAA	-	-	6
		RV-R1	TCIGGIARYTTCCASYACCAICC			
	Nested PCR	RV-F2	ACCRASTACTTTGGGTRWCCGTG	5'NCR-VP4/VP2	110bp	
		RV-R2	CTGTGTTGAWACYTGAGCICCCA			
HAdV	PCR	ADV-F	GCCSCARTGGKCWTACATGCACATC	Hexon	301bp	7
		ADV-R	CAGCACSCCICGRATGTCAAA			
HCoV	RT-PCR	hCoV-F	GGTTGGGACTATCCTAAGTGTGA	POL	440bp	8
		hCoV-R	CCATCATCAGATAGAATCATCATA			
HMPV	RT-PCR	MPV P-F	TyAACATTGCwACAGCAGGACC	P	247 bp	-
		MPV P-R	CTTCWGATTCWCCRCTTGTGCT			
HPIV	RT-PCR	PIV13-F	AGGWTGYSMRGATATAGGRAARTCAT	HA	HPIV-1: 439bp,<br>HPIV-2: 297bp,<br>HPIV-3: 390 bp,<br>HPIV-4: 174 bp.	6
		PIV13-R	CTWGTATATATATRTAGATCTTKTTRCCTAGT			
		PIV2-F	TAATTCCTCTTAAAATTGACAGTATCGA			
		PIV4-F	ATCCAGARRGACGTCACATCAACTCAT			
		PIV24-R	TRAGRCCMCCATAYAMRGGAAATA			
	Nested PCR	PIV13-F2	ACGACAAYAGGAARTCATGYTCT			
		PIV1-R	GACAACAATCTTTGGCCTATCAGATA			
		PIV3-R	GAGTTGACCATCCTYCTRTCTGAAAAC			
		PIV24-F	CYMAYGGRTGYAYTMGAATWCCATCATT			
		PIV2-R	GCTAGATCAGTTGTGGCATAATCT			
		PIV4-R	TGACTATRCTCGACYTTRAAATAAGG			
IFV	RT-PCR	FluAC1-F	GAACTCRTYCYWWATSWCAAWGRRGAAAT	NP	IFV-A: 301 bp,<br>IFV-B: 226 bp,<br>IFV-C: 111 bp	9
		FluB1-F	ACAGAGATAAAGAAGAGCGTCTACAA			
		FluABC2-R	ATKGCGCWYRAYAMWCTYARRTCTTCAWAIGC			
	Nested PCR	FluAB3-F	GATCAAGTGAKMGRRAGYMGRAAYCCAGG	NP		
		FluC3-F	AAATTGGAATTTGTTCCTTTCAAGGGACA			
		FluAC4-R	TCTTCAWATGCARSWSMAWKGCATGCCATC			
		FluB4-R	CTTAATATGGAAACAGGTGTTGCCATATT			
	RT-PCR	FluA -F	GACCAATCCTGTCACCTCTGAC	M	IFV-A: 210 bp,<br>IFV-B: 504 bp,<br>H5: 219bp,	
		FluA -R	AGCTGAGTGCGACCTCCTTAG			
		FluB -F	GGGACATGAACAACAAAGATGC	NS		

		FluB -R	TGTCAGCTATTATGGAGCTG		N1: 615bp,<br>H7: 184 bp,<br>H9: 383 bp,<br>N2: 281 bp,<br>N7: 282 bp.	
RSV	RT-PCR	RSVAB1-F	ATGGAGYTGCYRATCCWCARRRCAARTGCAAT	F	RSV-A: 363 bp,<br>RSV-B: 611 bp.	
		RSVAB2-R	AGGTGTWGTTACACCTGCATTRACACTRAATTC			
	Nested PCR	RSVA3-F	TTATACACTCAACAATRCCAAAAAWACC	F		
		RSVA4-R	AAATTCCCTGGTAATCTCTAGTAGTCTGT			
		RSVB3-F	ATCTTCCTAACTCTTGCTRTTAATGCATTG			
		RSVB4-R	GATGCGACAGCTCTGTTGATTTACTATG			

Supplementary Table 6-2. Primers and sequence information used for real-time PCR method for characterizing respiratory viruses.

Primers	Sequence (5’-3’)	3' Label	5' Label	Gene amplification	Reference
FluA-F	GACCRATCCTGTCACCTCTGAC	TAMRA	FAM	M2	10
FluA-R	AGGGCATTYTGGACAAAKCGTCTA				
FluA-P	TGCAGTCCTCGCTCACTGGGCACG				
09FluH1-F	GTGCTATAAACACCAGCCTYCCA	TAMRA	FAM	HA	10
09FluH1-R	CGGGATATTCCTTAATCCTGTRGC				
09FluH1-P	CAGAATATACA“T”~~T~~CCRGTCACAATTGGARAA				
FluB-F	TGCCTACCTGCTTTMMYTRACA	TAMRA	FAM	M	11
FluB-R	CCRAACCAACARTGTAATTTTTCTG				
FluB-P	TGCTTTGCCTTCTCCA				
RSVA-F	GCTCTTAGCAAAGTCAAGTTGAATGA	TAMRA	FAM	N	12
RSVA-R	TGCTCCGTTGGATGGTGTATT				
RSVA-P	ACACTCAACAAAGATCAACTTCTGTCATCCAGC				
RSVB-F	GCTCTTAGCAAAGTCAAGTTGAATGA	TAMRA	FAM	N	12
RSVA-R	TGCTCCGTTGGATGGTGTATT				
RSVA-P	ACACTCAACAAAGATCAACTTCTGTCATCCAGC				
229E-F	CAGTCAAATGGGCTGATGCA	TAMRA	FAM	NP	13
229E-R	AAAGGGCTATAAAGAGAATAAGGTATTCT				
229E-P	CCCTGACGACCACGTTGTGGTTCA				
NL63-F	GACCAAAGCACTGAATAACATTTTCC	TAMRA	FAM	NP	13
NL63-R	ACCTAATAAGCCTCTTTCTCAACCC				
NL63-P	AACACGCTTCCAACGAGGTTTCTTCAACTGAG				
OC43-F	CGATGAGGCTATTCCGACTAGGT	TAMRA	FAM	NP	13
OC43-R	CCTTCCTGAGCCTTCAATATAGTAACC				
OC43-P	TCCGCCTGGCACGGTACTCCCT				
HKU1-F	CCTTGCGAATGAATGTGCT	TAMRA	FAM	Replicase1b	13
HKU1-R	TTGCATCACCACTGCTAGTACCAC				
HKU1-P	TGTGTGGCGGTTGCTATTATGTTAAGCCTG				
hPIV1-F	TGATTTAAACCCGGTAATTTCTCAT	TAMRA	FAM	HN (Hemagglutinin neuraminidase)	14
hPIV1-R	CCTTGTTCCTGCAGCTATTACAGA				
hPIV1-P	ACGACAACAGGAAATC				
hPIV2-F	AGGACTATGAAAACCATTTACCTAAGTGA	TAMRA	FAM	HN	14
hPIV2-R	AAGCAAGTCTCAGTTCAGCTAGATCA				
hPIV2-P	ATCAATCGCAAAAGCTGTTCAGTCACTGCTATAC				
hPIV3-F	TGATGAAAGATCAGATTATGCATCATC	TAMRA	FAM	HN	14
hPIV3-R	CCGGGACACCCAGTTGTG				
hPIV3-P	TGGACCAGGGATATACTACAAAGGCAAAATAATATTTCTC				
hPIV4-F	CAAAYGATCCACAGCAAAGATTC	TAMRA	FAM	Nucleocapsid	14
hPIV4-R	ATGTGGCCTGTAAGGAAAGCA				
hPIV4-P	GTATCATCATCTGCCAAATCGGCAATTAAACA				
HBoV-F	AGAGGCTCGGGCTCATATCA	TAMRA	FAM	NP-1	15
HBoV-R	CACTTGGTCTGAGGTCTTCGAA				
HBoV-P	AGGAACACCCAATCARCCACCTATCGTCT				
hAdV-F	GCCACGGTGGGGTTTCTAAACTT	TAMRA	FAM	Hexon	16
hAdV-R	GCCCCAGTGGTCTTACATGCACATC				
hAdV-P	TGCACCAGACCCGGGCTCAGGTACTCCGA				
hMPV-F	CATAYAARCATGCTATATTAAAAGAGTCTC	TAMRA	FAM	NP	17
hMPV-R	CCTATYTCTGCAGCATATTTGTAATCAG				
hMPV-P	TGYAATGATGARGGTGTCACTGCRGTTG				
RHV-F1	CTTTGAGTCCTCCGGCCC	BHQ1	FAM	5’UTR	-
RHV-R1	CCCGCAATTGCTCATTACGAC				
RHV-P1	TGAATGTGGCTAACCTTAACCCTGCAGC				
RHV-F2	CCCCACTGGCGACAGTGT	BHQ1	FAM	5’UTR	-
RHV-R2	GGGGCTCTTCACACCTTGTC				
RHV-P2	CTAGCCTGCGTGGCTGCCTGC				
RHV-F3	CCACTAGTTTGGTCGATGAGGCT	BHQ1	FAM	5’UTR	-
RHV-R3	TGGCCGCCACGCAGG				
RHV-P3	GAATTCCCCACGGGCGACCGTGTC				
RHV-F4	TGTGAAGAGCCCCGTGTGCT	MGB	FAM	5’UTR	-
RHV-R4	TGCAGGGTTAAGGTTAGCCACAT				
RHV-P4	CAGGGGCCGGAGGAC				

B =C, G, or T; H =A, C, or T; R= A or G; S =G or C; Y =C or T.

Supplementary Table 6-3. Primers and sequence information used for characterizing respiratory bacteria.

**Bacteria**	**Gene amplification**	**The direction of primers**	**Sequence (5'-3')**	**Amplicon size (bp)**
S. pneumoniae	*nuc*	Forward primer	GCGATTGATGGTGATACGGTT	278
		Backward primes	AGCCAAGCCTTGACGAACTAAAGC	
M. pneumoniae	*ATPase operon*	Forward primer	GAAGCTTATGGTACAGGTTGG	144
		Backward primes	ATTACCATCCTTGTTGTAAGG	
C. pneumoniae	16s	Forward primer	TGACAACTGTAGAAATACAGC	465
		Backward primes	CGCCTCTCTCCTATAAAT	
K. pneumoniae	*bla*	Forward primer	AAgATCCACTATCgCCAgCAgg	-
		Backward primes	ATTCAgTTCCgTTTCCCAgCgg	
GAS	*speB*	Forward primer	GTCAACATGCAGCTACAGGA	257
		Backward primes	AATACCAACATCAGCCATCA	
P. aeruginosa	toxA	Forward primer	GACAACGCCCTCAGCATCACCAGC	396
		Backward primes	CGCTGGCCCATTCGCTCCAGCGCT	
L. pneumophila	16s	Forward primer	AAGATTAGCCTGCGTCCGA	654
		Backward primes	GTCAACTTATCGCGTTTGCT	

Supplemental Reference

1 Kim, H. J., Fay, M. P., Feuer, E. J. & Midthune, D. N. Permutation tests for joinpoint regression with applications to cancer rates. *Statistics in medicine* **19**, 335-351, doi:10.1002/(sici)1097-0258(20000215)19:3<335::aid-sim336>3.0.co;2-z (2000).

2 https://surveillance.cancer.gov/help/joinpoint/setting-parameters/method-and-parameters-tab/apc-aapc-tau-confidence-intervals/average-annual-percent-change-aapc.

3 Price, O. H., Sullivan, S. G., Sutterby, C., Druce, J. & Carville, K. S. Using routine testing data to understand circulation patterns of influenza A, respiratory syncytial virus and other respiratory viruses in Victoria, Australia. *Epidemiology and infection* **147**, e221, doi:10.1017/s0950268819001055 (2019).

4 Woolf, B. On estimating the relation between blood group and disease. *Annals of human genetics* **19**, 251-253, doi:10.1111/j.1469-1809.1955.tb01348.x (1955).

5 Kapoor, A. *et al.* Human bocaviruses are highly diverse, dispersed, recombination prone, and prevalent in enteric infections. *J Infect Dis* **201**, 1633-1643, doi:10.1086/652416 (2010).

6 Coiras, M. T., Aguilar, J. C., García, M. L., Casas, I. & Pérez-Breña, P. Simultaneous detection of fourteen respiratory viruses in clinical specimens by two multiplex reverse transcription nested-PCR assays. *Journal of medical virology* **72**, 484-495, doi:10.1002/jmv.20008 (2004).

7 Allard, A., Albinsson, B. & Wadell, G. Detection of adenoviruses in stools from healthy persons and patients with diarrhea by two-step polymerase chain reaction. *Journal of medical virology* **37**, 149-157, doi:10.1002/jmv.1890370214 (1992).

8 Woo, P. C. *et al.* Characterization and complete genome sequence of a novel coronavirus, coronavirus HKU1, from patients with pneumonia. *Journal of virology* **79**, 884-895, doi:10.1128/jvi.79.2.884-895.2005 (2005).

9 Coiras, M. T., Pérez-Breña, P., García, M. L. & Casas, I. Simultaneous detection of influenza A, B, and C viruses, respiratory syncytial virus, and adenoviruses in clinical samples by multiplex reverse transcription nested-PCR assay. *Journal of medical virology* **69**, 132-144, doi:10.1002/jmv.10255 (2003).

10 WHO: CDC protocol of realtime RTPCR for influenza A (H1N1), 30 April 2009.

11 Chidlow, G. R., Harnett, G. B., Shellam, G. R. & Smith, D. W. An economical tandem multiplex real-time PCR technique for the detection of a comprehensive range of respiratory pathogens. *Viruses* **1**, 42-56, doi:10.3390/v1010042 (2009).

12 Hu, A., Colella, M., Tam, J. S., Rappaport, R. & Cheng, S. M. Simultaneous detection, subgrouping, and quantitation of respiratory syncytial virus A and B by real-time PCR. *Journal of clinical microbiology* **41**, 149-154, doi:10.1128/jcm.41.1.149-154.2003 (2003).

13 Dare, R. K. *et al.* Human coronavirus infections in rural Thailand: a comprehensive study using real-time reverse-transcription polymerase chain reaction assays. *J Infect Dis* **196**, 1321-1328, doi:10.1086/521308 (2007).

14 van de Pol, A. C. *et al.* Increased detection of respiratory syncytial virus, influenza viruses, parainfluenza viruses, and adenoviruses with real-time PCR in samples from patients with respiratory symptoms. *Journal of clinical microbiology* **45**, 2260-2262, doi:10.1128/jcm.00848-07 (2007).

15 Lu, X. *et al.* Real-time PCR assays for detection of bocavirus in human specimens. *Journal of clinical microbiology* **44**, 3231-3235, doi:10.1128/jcm.00889-06 (2006).

16 Heim, A., Ebnet, C., Harste, G. & Pring-Akerblom, P. Rapid and quantitative detection of human adenovirus DNA by real-time PCR. *Journal of medical virology* **70**, 228-239, doi:10.1002/jmv.10382 (2003).

17 Maertzdorf, J. *et al.* Real-time reverse transcriptase PCR assay for detection of human metapneumoviruses from all known genetic lineages. *Journal of clinical microbiology* **42**, 981-986, doi:10.1128/jcm.42.3.981-986.2004 (2004).
