# Supplementary material for: Etiological and epidemiological features of acute respiratory infections in China
Source: Nat Commun. 2021 Aug 18;12:5026. doi: 10.1038/s41467-021-25120-6 (PMC8373954; doi:10.1038/s41467-021-25120-6)
Supplement: Supplementary file 3 — Reporting Summary [file 41467_2021_25120_MOESM3_ESM.pdf]

nature portfolio

Corresponding author(s): Li-Ping Wang, Li-Qun Fang, Wei Liu

Last updated by author(s): Jun 17, 2021

Reporting Summary

Nature Portfolio wishes to improve the reproducibility of the work that we publish. This form provides structure for consistency and transparency in reporting. For further information on Nature Portfolio policies, see our Editorial Policies and the Editorial Policy Checklist.

Statistics

For all statistical analyses, confirm that the following items are present in the figure legend, table legend, main text, or Methods section.

n/a	Confirmed	
☐	☒	The exact sample size (*n*) for each experimental group/condition, given as a discrete number and unit of measurement
☒	☐	A statement on whether measurements were taken from distinct samples or whether the same sample was measured repeatedly
☐	☒	The statistical test(s) used AND whether they are one- or two-sided<br>*Only common tests should be described solely by name; describe more complex techniques in the Methods section.*
☐	☒	A description of all covariates tested
☐	☒	A description of any assumptions or corrections, such as tests of normality and adjustment for multiple comparisons
☐	☒	A full description of the statistical parameters including central tendency (e.g. means) or other basic estimates (e.g. regression coefficient) AND variation (e.g. standard deviation) or associated estimates of uncertainty (e.g. confidence intervals)
☐	☒	For null hypothesis testing, the test statistic (e.g. *F*, *t*, *r*) with confidence intervals, effect sizes, degrees of freedom and *P* value noted<br>*Give P values as exact values whenever suitable.*
☒	☐	For Bayesian analysis, information on the choice of priors and Markov chain Monte Carlo settings
☒	☐	For hierarchical and complex designs, identification of the appropriate level for tests and full reporting of outcomes
☒	☐	Estimates of effect sizes (e.g. Cohen's *d*, Pearson's *r*), indicating how they were calculated

Our web collection on statistics for biologists contains articles on many of the points above.

Software and code

Policy information about availability of computer code

Data collection	No software was used in the data collection.
Data analysis	R statistical software (version 3.6.3, R Development Core Team 2020) was used in the data analysis. The Join-Point regression (JPR) model was used to describe trends that were not constant over ages (V.4.7.0.0, Statistical Research and Applications Branch, National Cancer Institute, USA). No custom code was used in this analyses.

For manuscripts utilizing custom algorithms or software that are central to the research but not yet described in published literature, software must be made available to editors and reviewers. We strongly encourage code deposition in a community repository (e.g. GitHub). See the Nature Portfolio guidelines for submitting code & software for further information.

Data

Policy information about availability of data

All manuscripts must include a data availability statement. This statement should provide the following information, where applicable:

- Accession codes, unique identifiers, or web links for publicly available datasets
- A description of any restrictions on data availability
- For clinical datasets or third party data, please ensure that the statement adheres to our policy

Relevant data that support the findings of this study and model results generated as part of this study are publicly available within the paper and its Supplementary Information Files. Raw data are not publicly available and are protected due to data privacy laws, which were used under license for the current study, but are available upon reasonable request to the corresponding author and with permission from the data provider (Li-Ping Wang). The request will be responded within one week. Source data are provided with the paper.

Field-specific reporting

Please select the one below that is the best fit for your research. If you are not sure, read the appropriate sections before making your selection.

- [x] Life sciences
- [ ] Behavioural & social sciences
- [ ] Ecological, evolutionary & environmental sciences

For a reference copy of the document with all sections, see nature.com/documents/nr-reporting-summary-flat.pdf

Life sciences study design

All studies must disclose on these points even when the disclosure is negative.

Sample size	231107 patients with acute respiratory infections. The sample size of each sentinel hospital was determined in advance by the standard operating protocol (SOP) of surveillance. The number of sentinel hospitals was determined in proportional to the total population size by province and sample size of each hospital was additionally based on high-coverage of medical service, adequate capacity for surveillance and laboratory testing.
Data exclusions	Patients with confirmed diagnosis of non-infectious respiratory diseases such as asthma and respiratory tumor were excluded. Another 1930 patients were excluded due to incomplete data records or for having an inadequate sample volume.
Replication	Reproducibility of experimental findings was not applicable because our study is observational.
Randomization	Randomization used to allocate participates into experimental groups was not applicable because our study is observational.
Blinding	Blinding was not applicable because our study is observational.

Reporting for specific materials, systems and methods

We require information from authors about some types of materials, experimental systems and methods used in many studies. Here, indicate whether each material, system or method listed is relevant to your study. If you are not sure if a list item applies to your research, read the appropriate section before selecting a response.

Materials & experimental systems

n/a	Involved in the study	
x		Antibodies
x		Eukaryotic cell lines
x		Palaeontology and archaeology
x		Animals and other organisms
	x	Human research participants
x		Clinical data
x		Dual use research of concern

Methods

n/a	Involved in the study	
x		ChIP-seq
x		Flow cytometry
x		MRI-based neuroimaging

Human research participants

Policy information about studies involving human research participants

Population characteristics	Totally 231107 cases from 277 sentinel hospitals and 92 reference laboratories in 106 cities were available for the final analyses. Their median age was 14 years (IQR 2–44 years, 52.2% were aged <18 years), 53.1% were hospitalized, and 20.2% were diagnosed as pneumonia.
Recruitment	Samples were recruited from patients with acute respiratory infections (ARI) administered in 277 sentinel hospitals of all 31 provinces (autonomous regions or municipalities) on the Chinese mainland. In this study, a case of ARI was defined as: (1) at least one of the following conditions: fever, abnormal white blood cell differentials, leukocytosis or leukopenia, and (2) at least one of the following symptoms/signs: cough, chills, expectoration, nasal congestion, sore throat, chest pain, tachypnea, and abnormal pulmonary breath sounds. The number of sentinel hospitals from Western China were less than those in the densely populated regions, as there was a lower than average population size in this region. This limitation may have hindered a full understanding of the dynamic pattern of the ARIs in this region.
Ethics oversight	All participating hospitals and laboratories used a standard operating protocol (SOP) of surveillance that included guidelines for patient’s enrollment, specimen’s collection, laboratory testing, data recording and management, developed by China CDC. The SOP was approved by the institutional review board at each institution and at the center for disease control and prevention. The surveillance protocol was reviewed and approved by the ethics review committees of the China CDC (2015-025).

Note that full information on the approval of the study protocol must also be provided in the manuscript.
